# Overview of SERPING1 Variations Identified in Hungarian Patients With Hereditary Angioedema

**DOI:** 10.3389/falgy.2022.836465

**Published:** 2022-03-17

**Authors:** Edina Szabó, Dorottya Csuka, Noémi Andrási, Lilian Varga, Henriette Farkas, Ágnes Szilágyi

**Affiliations:** ^1^Department of Internal Medicine and Hematology, Semmelweis University, Budapest, Hungary; ^2^Hungarian Angioedema Center of Reference and Excellence, Semmelweis University, Budapest, Hungary; ^3^Second Department of Pediatrics, Semmelweis University, Budapest, Hungary

**Keywords:** C1-inhibitor, C1-INH-HAE, hereditary angioedema, long-range PCR, MLPA, mutation, sequencing, SERPING1

## Abstract

**Background:**

Hereditary angioedema (HAE) due to C1-inhibitor (C1-INH) deficiency (C1-INH-HAE) is a rare autosomal dominant disorder, characterized by recurrent, unpredictable edematous symptoms involving subcutaneous, and/or submucosal tissue. C1-INH-HAE may be caused by more than 700 different mutations in the gene encoding C1-INH (*SERPING1*) that may lead to decreased protein synthesis or to functional deficiency.

**Methods:**

Concentrations of C1-INH, C4, C1q, and anti-C1-INH antibodies, as well as functional C1-INH activity were determined in subjects suffering from edematous symptoms and admitted to the Hungarian Angioedema Center of Reference and Excellence. In those patients, who were diagnosed with C1-INH-HAE based on the complement measurements, *SERPING1* was screened by bidirectional sequencing following PCR amplification and multiplex ligation-dependent probe amplification. For detecting large deletions, long-range PCRs covering the entire *SERPING1* gene by targeting 2–7 kb long regions were applied.

**Results:**

Altogether 197 individuals with C1-INH deficiency belonging to 68 families were identified. By applying Sanger sequencing or copy number determination of *SERPING1* exons, 48 different mutations were detected in 66/68 families: 5 large and 15 small insertions/deletions/delins, 16 missense, 6 nonsense, and 6 intronic splice site mutations. Two novel variations (p.Tyr199Ser [c.596A>C] and the duplication of exon 7) were shown to cosegregate with deficient C1-inhibitor level and activity, while two other variations were detected in single patients (c.797_800delinsCTTGGAGCTCAAGAACTTGGAGCT and c.812dup). A series of long PCRs was applied in the remaining 2 families without an identified mutation and a new, 2606 bp long deletion including the last 91 bp of exon 6 (c.939_1029+2515del) was identified in all affected members of one pedigree. In the remaining one family, a deep intronic *SERPING1* variation (c.1029+384A>G) was detected by a targeted next-generation sequencing panel as reported previously.

**Conclusions:**

Sequencing and copy number determination of *SERPING1* exons uncover most pathogenic variants in C1-INH-HAE patients, and further methods are worth to be applied in cases with unrevealed genetic background. Since knowledge of the genetic background may support the establishment of the correct and early diagnosis of C1-INH-HAE, identification of causative mutations and reporting data supporting the interpretation on the pathogenicity of these variants is of utmost importance.

## Introduction

Hereditary angioedema (HAE) due to C1-inhibitor (C1-INH) deficiency (C1-INH-HAE) is a rare autosomal dominant disorder, characterized by recurrent, unpredictable, nonpitting edematous symptoms involving the subcutaneous and/or submucosal tissues and showing intra- and interindividual variability. C1-INH-HAE episodes usually affect the extremities (arms, hands, feet, and legs), the face, lips, eyelids, bowels, and genitalia. In those rare cases when the edema evolves in the upper airways, the disease may lead to a potentially life-threatening condition within hours, without the proper treatment. Since the first pathogenic *SERPING1* mutation was described in 1987 (1), the presence of C1-INH-HAE was explained by more than 700 different mutations (2–4) in the gene encoding C1-INH (*SERPING1*) that can either cause decreased protein synthesis (C1-INH-HAE type I) or functional deficiency (C1-INH-HAE type II). The mutations in the background of C1-INH-HAE type II are usually missense mutations affecting the reactive center loop of C1-INH encoded in exon 8. On the other hand, variations leading to the development of C1-INH-HAE type I are quite heterogeneous and are distributed over the exons and introns of the entire *SERPING1* gene, in form of deletions or insertions of various sizes along with missense or nonsense substitutions, leading to the defect of C1-INH synthesis or secretion.

Molecular genetic testing is not considered as obligatory to confirm the diagnosis of C1-INH-HAE, though analyzing the segregation of novel *SERPING1* variants in case of available family members is recommended to confirm the pathogenicity and penetrance (5). Considering the facts that (1) *SERPING1* variants are predominantly associated with decreased functional C1-INH activity and that (2) C1-INH-HAE is inherited in an autosomal dominant pattern with high penetrance, novel *SERPING1* mutations need only in a few cases to be functionally characterized *in vitro*, when considering their pathogenicity (6, 7).

Establishing the correct and early diagnosis of C1-INH-HAE (including the screening of all symptomatic and asymptomatic first-degree relatives of the diagnosed patients) is of high importance for the affected subjects' proper treatment, prognosis, and quality of life. Furthermore, previously asymptomatic family members may – in the future – suffer unpredictable symptoms that require specific treatment. Knowledge of a pedigree's causative *SERPING1* mutation is also useful as this information may contribute to preimplantation and prenatal diagnosis or underpin diagnosis in cases with uncertain complement results that may occur in very early childhood (8).

Here, we explain our strategy on genetic work-up exploring the *SERPING1* gene and provide an overview of *SERPING1* mutations identified in Hungarian C1-INH-HAE patients over the past decades involving those published previously as well as new families with novel mutations.

## Materials and Methods

In the Hungarian Angioedema Center of Reference and Excellence, 197 individuals (110 female, 87 male, mean age 42.8 years) belonging to 68 families were identified with C1-INH deficiency. The diagnosis of C1-INH-HAE was established according to the international consensus criteria (9), based on the following complement measurements: serum concentration of C1-INH was determined by radial immunodiffusion (10), C4 level by immunoturbidimetry (Beckman Coulter, Brea, CA, USA), levels of anti-C1-INH antibodies and C1q were measured by ELISA (11, 12), whereas the functional C1-INH activity was analyzed by using a commercial kit (Quidel, San Diego, CA, USA) in the serum samples of subjects suffering from edematous symptoms.

In those patients, who were diagnosed with C1-INH-HAE based on the complement measurements, and also in their available family members, genomic DNA was isolated from peripheral or umbilical cord blood samples by the salting-out method (13).

Bidirectional DNA sequencing following PCR amplification was applied to screen the whole coding region of the gene encoding C1-inhibitor (*SERPING1*; OMIM #606860). Amplification of genomic DNA was carried out in 35 cycles with GoTaq G2 DNA polymerase (Promega, Madison, WI, USA) according to the manufacturer's instructions (details of the reactions are available upon request) applying the primers listed in Table 1. Before sequencing, PCR products were purified with Exonuclease I and FastAP Thermosensitive Alkaline Phosphatase (Thermo Scientific, Waltham, MA, USA) and sequencing was performed using the BigDye Terminator v3.1 Cycles Sequencing Kit (Life Technologies, Carlsbad, CA, USA) according to the manufacturer's instructions with the sequencing primers specified in Table 1. After sodium acetate/ethanol purification, sequencing products were separated with an Applied Biosystems 3130xl Genetic Analyzer (Life Technologies, Carlsbad, CA, USA).

**Table 1:** Primer sequences and PCR conditions applied in this study.

	**Studied exon(s)**	**Primer**	**Product size (bp)**	**Applied enzyme**	**T_**m**_**
Primers for PCR	1–2	F: 5' TTGAGGAATAACGGAGGTGAG 3' R: 5' AGGAGGAGTAGGCTGAGAAAA 3'	1,278	GoTaq	59°C
	3	F: 5' GTACTAGCCAAGCAAGTGAGTC 3' R: 5' AGCAATCGTGCCTATTACATC 3'	1,082	GoTaq	59°C
	4	F*: 5' ATACCCTCCATTCCAGCCTGGTC 3' R*: 5' CTTCACCTGCTCTGCAGTCCATC 3'	368	GoTaq	59°C
	5–6	F: 5' CACCATGCCGTATTCACTAA 3' R: 5' AGGGTGGAAATACAGATGGAAG 3'or R: 5' TCCCTCCCTACTCATCAAAC 3'	798	GoTaq	59°C
	7	F: 5' TCAGTGGTGGAGTCAGGGTA 3' R: 5' CCAATGGGATAATAGCACCTAC 3'	631	GoTaq	59°C
	8	F: 5' CTGCCAGAGGGTACAGTATGT 3' R: 5' GAGATGGGAGGATTGTTTGA 3'	823	GoTaq	59°C
Primers for sequencing	1–2	F: 5' CCCCGTTCACCCCACCTACCA 3' or F: 5' TTGAGGAATAACGGAGGTGAG 3' R*: 5' GCCTGAAGGGTTAATCCTCAGCCA 3'			
	3	F: 5' TGGTGGTGGTTCTAAGACAGATT 3' R: 5' AGAGGCATGGCTTTGTAAGTG 3'			
	4	F*: 5' ATACCCTCCATTCCAGCCTGGTC 3' R*: 5' CTTCACCTGCTCTGCAGTCCATC 3'			
	5–6	F: 5' CTCAAATCGTGCTCATGGAA 3' R: 5' AGGGTGGAAATACAGATGGAAG 3' or R: 5' TCCCTCCCTACTCATCAAAC 3'			
	7	F: 5' TCAGTGGTGGAGTCAGGGTA 3' R: 5' CCAATGGGATAATAGCACCTAC 3'			
	8	F: 5' GGCAAACAAGGGAAGAGGAAG 3' R: 5' AGCCTGGGTGACAGATTGAGA 3'			
Primers for long PCR	1–3	F: 5' TGCACTGGAGCTGCCTGGTGA 3' R: 5' AGAGGCATGGCTTTGTAAGTG 3'	2,777	GoTaq	60°C
	3–5	F: 5' TGGTGGTGGTTCTAAGACAGATT 3' R: 5' GGAGGGTTGCTCTAATGCAG 3'	6,676	Phusion Flash	58°C
	5–7	F: 5' CACCATGCCGTATTCACTAA 3' R: 5' CCAATGGGATAATAGCACCTAC 3'	6,209	Phusion Flash	60°C
	7–8	F: 5' TCAGTGGTGGAGTCAGGGTA 3' R: 5' CACAGGGGTCAGAATCACCT 3'	5,289	Phusion Flash	62°C
	8	F: 5' GGCAAACAAGGGAAGAGGAAG 3' R: 5' TGCTAAAAACACCCTCCAAA 3'	6,310	Phusion Flash	60°C
Primers for the verification of exon 7 duplication	exon 7 MLPA probe hybridization site	F: 5' TACCAGGATCACCAAACTCAGAT 3' R: 5' CACAATCTGAGTTTGGTGATCCTG 3'		Phusion Flash	64°C

* *Based on primer sequences published in (14)*.

*F, forward; R, reverse*.

In order to detect large deletions or duplications in the *SERPING1* gene, multiplex ligation-dependent probe amplification (MLPA) was performed applying the SALSA MLPA P243-A3 or P243-B1 SERPING1-F12 probemixes (MRC Holland, Amsterdam, The Netherlands). Data were analyzed using the Coffalyser.Net™ MLPA analysis software (MRC Holland, Amsterdam, The Netherlands) according to the manufacturers' instructions.

A series of long-range PCRs amplifying 2–7 kb sequences was performed with Promega GoTaq G2 DNA polymerase (Promega, Madison, WI, USA) or Phusion Flash PCR Master Mix (Thermo Scientific, Waltham, MA, USA) in cases of unresolved genetic background and their family members using the primers listed in Table 1.

The identified genetic variations were named according to the Human Genomic Variation Society (HGVS) recommendations (15) and for cDNA nucleotide numbering the reference sequence of *SERPING1* (ID: NM_000062.3) was used. Interpretation of sequence variants was based on the criteria established by the American College of Medical Genetics and Genomics (ACMG) (16). The possible functional effect of an identified novel rare variation was assessed using *in silico* prediction tools, such as Sorting Intolerant From Tolerant (SIFT) (http://siftdna.org/www/Extended SIFT chrcoords submit.html)(17), PolyPhen (version 2) (http://genetics.bwh.harvard.edu/pph2/) (18), PROVEAN (http://provean.jcvi.org/genomesubmit.php) (19), CADD (https://cadd.gs.washington.edu/) (20), Mutation-Taster (http://mutationtaster.org) (21), or Human Splicing Finder (22) (version 3.1; http://www.umd.be/HSF3/~[21]).

## Results

**Figure 1:**
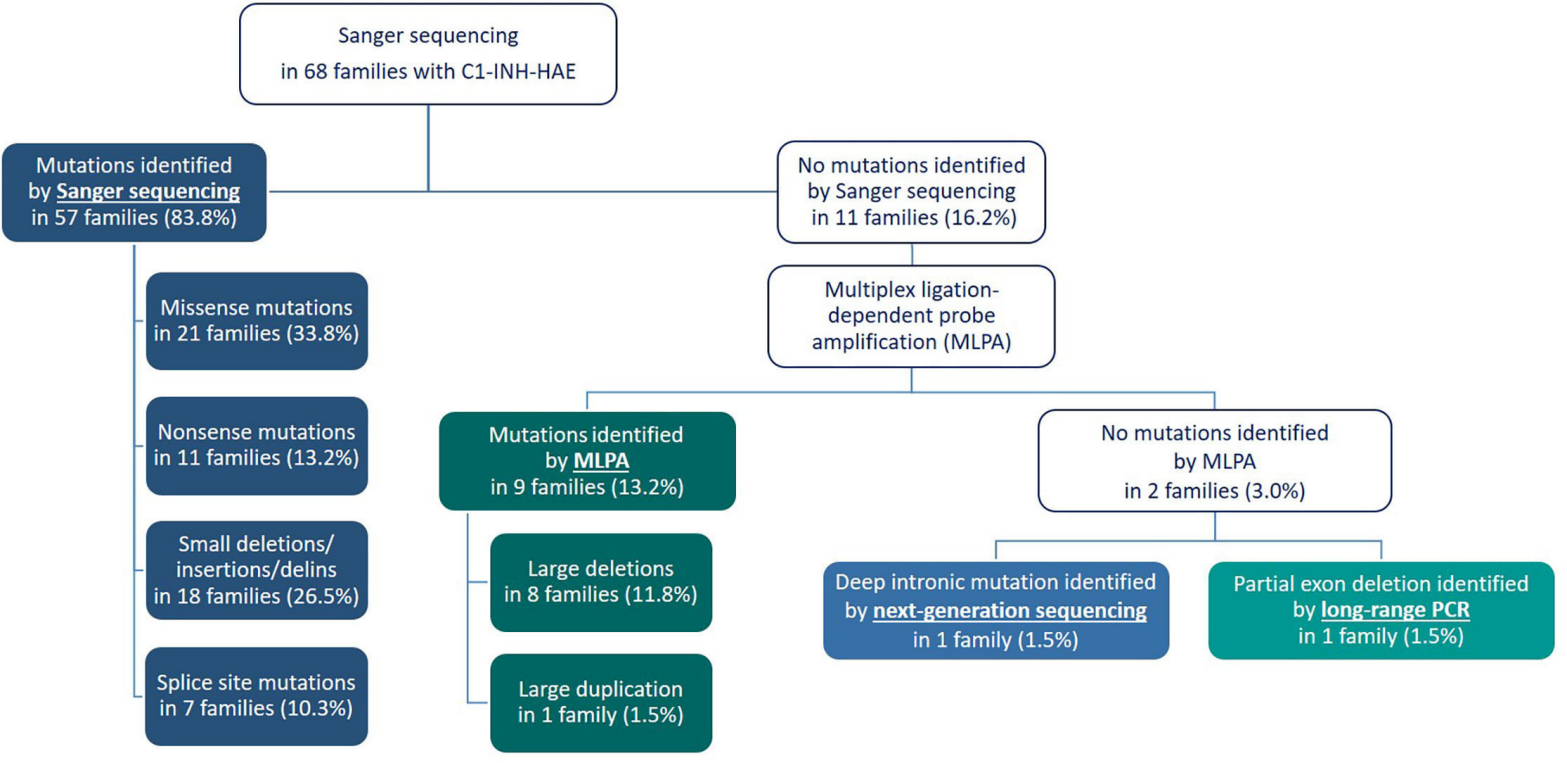
Flow chart illustrating the algorithm for molecular genetic testing in the Hungarian Angioedema Center of Reference and Excellence.

By applying Sanger sequencing to screen coding exons and exon–intron boundaries of the *SERPING1* gene, 43 different mutations were identified in 57 of the studied 68 families (Figure 1). Alterations changing the coding region of *SERPING1* included 10 small deletions, 4 small duplications, 1 delins, and 22 substitutions, the latter resulting in 16 missense and 6 nonsense variations, while 6 mutations influenced the intronic splice sites (Tables 2–5). All the cases (13 patients in 5 families) with C1-INH-HAE type II carried the p.Arg466Cys missense variation.

**Table 2:** Missense and nonsense mutations identified in the *SERPING1* gene.

**Affected exon**	**cDNA position**	**Protein position**	**Number of affected families**	**Number of affected patients**	**References**
2	c.1A>G	p.Met1Val	1	2	(23)
3	c.65C>G	p.Ser22*	1	2	(7)
3	c.94C>T	p.Gln32*	3	12	(24)
3	c.253G>T	p.Glu85*	1	1	(3)
3	c.389G>A	p.Cys130Tyr	2	6	(24)
3	c.425T>C	p.Leu142Ser	1	3	(25)
3	c.503C>A	p.Ala168Asp	1	2	(26)
4	c.553G>C	p.Ala185Pro	1	6	(3)
4	c.596A>C	p.Tyr199Ser	1	3	-
4	c.667C>T	p.Gln223*	1	3	(24)
5	c.728T>C	p.Leu243Pro	1	1	(27)
5	c.752T>G	p.Leu251Arg	1	2	(3)
6	c.911A>G	p.Asp304Gly	1	3	(3)
6	c.988T>G	p.Tyr330Asp	1	6	(25)
7	c.1180A>C	p.Thr394Pro	1	1	(28)
7	c.1223A>T	p.Asp408Val	1	1	(24)
8	c.1396C>T	p.Arg466Cys	5	13	(29)
8	c.1418T>A	p.Val473Glu	1	1	(24)
8	c.1423C>T	p.Gln475*	1	1	(3)
8	c.1478G>A	p.Gly493Glu	1	1	(30)
8	c.1480C>T	p.Arg494*	4	6	(28)
8	c.1493C>G	p.Pro498Arg	1	1	(24)

**Table 3:** Small deletions/insertions/delins identified in the *SERPING1* gene.

**Affected exon**	**cDNA position**	**Protein position**	**Number of affected families**	**Number of affected patients**	**References**
3	c.106_107del	p.Ser36Phefs*21	1	2	(28)
3	c.249del	p.Asp84Metfs*64	1	1	(31)
3	c.392_393del	p.Ser131*	1	1	(24)
3	c.435_476del	p.Leu146_Ala159del	4	26	(24)
5	c.705del	p.Phe236Leufs*2	1	4	(3)
5	c.797_800delinsCTTGGAGCTCAAGAACTTGGAGCT	p.Val266Alafs*20	1	1	-
5	c.812dup	p.Asn271Lysfs*34	1	1	-
6	c.982del	p.Lys328Argfs*13	1	1	(32)
7	c.1106del	p.Asn369Alafs*28	1	5	(24)
7	c.1127dup	p.Ser377Phefs*48	1	1	(33)
7	c.1147dup	p.Met383Asnfs*42	1	2	(3)
8	c.1356_1357del	p.Val454Glyfs*18	1	2	(33)
8	c.1357_1382dup	p.Ile462Glyfs*123	1	1	(24)
8	c.1391_1392del	p.Val464Glyfs*8	1	3	(33)
8	c.1466del	p.Pro489Leufs*87	1	2	(33)

**Table 4:** Intronic mutations identified in the *SERPING1* gene.

**Affected intron**	**cDNA position**	**Number of affected families**	**Number of affected patients**	**References**
1	c.51+1G>A	2	7	(23)
3	c.550+1G>A	1	3	(24)
3	c.550+2dup	1	5	(24)
3	c.550+5G>A	1	1	(7)
4	c.686-3C>G	1	12	(7)
5	c.889+1G>A	1	3	(2)
6	c.1029+384A>G	1	4	(34)

**Table 5:** Large deletions/duplication identified in the *SERPING1* gene.

**Affected exon**	**Variation**	**Number of affected families**	**Number of affected patients**	**References**
4	exon 4 deletion	5	9	(35)
6	c.939_1029+2515del	1	2	-
7	exon 7 deletion	1	4	(33)
7	exon 7 duplication	1	9	-
7–8	exon 7–8 deletion	1	2	(23)
1–8	exon 1–8 (whole gene) deletion	1	6	(35)

Among the detected substitutions, one novel (c.596A>C) was identified that causes a tyrosine to serine amino acid change at codon 199 (Tyr199Ser) located in the enzymatically active serpin domain. The potential effect of this missense variation was estimated by five different prediction programs, with 4 out of the 5 programs predicting the change to be disease causing and one to be neutral (SIFT: damaging [score: 0.049]; PROVEAN: deleterious [score: −5.03]; Mutation Taster: disease-causing [probability: 0.67]; CADD PHRED-like score: 23.1; Polyphen-2: benign [score: 0.250] [sensitivity: 0.91; specificity: 0.88] by the HumDiv model). Available family members were also tested for the carrier state of the mutation, and the affected sister and their mother were found to be heterozygous for this substitution and both of them showed impaired complement results (low C4 with deficient C1-INH function and level).

A novel single base duplication (c.812dup) was identified in a patient with low C1-INH level and activity. By the insertion of an adenine in exon 5, this mutation causes frameshift and introduces a premature stop codon (p.Asn271Lysfs^*^34) in the mRNA. Two of the applied prediction tools were suitable for estimating the potential effect of a duplication; both indicated a deleterious effect for this variation with high probability (Mutation Taster: disease-causing [probability: 1]; CADD PHRED-like score: 26.3). No other rare variation was detected in the patient, who had no available family members.

A complex variation with the deletion of 4 nucleotides and the insertion of 24 bp (c.797_800delinsCTTGGAGCTCAAGAACTTGGAGCT) was also identified that causes a frameshift and premature termination of protein synthesis at the amino acid position 266 (p.Val266Alafs^*^). The patient who carried this mutation did not have any other rare variation in the *SERPING1* gene and her complement measurements showed deficient C1-inhibitor level as well as below-normal C4 level. Segregation could not be verified in this case as no family members were available for testing.

**Figure 2:**
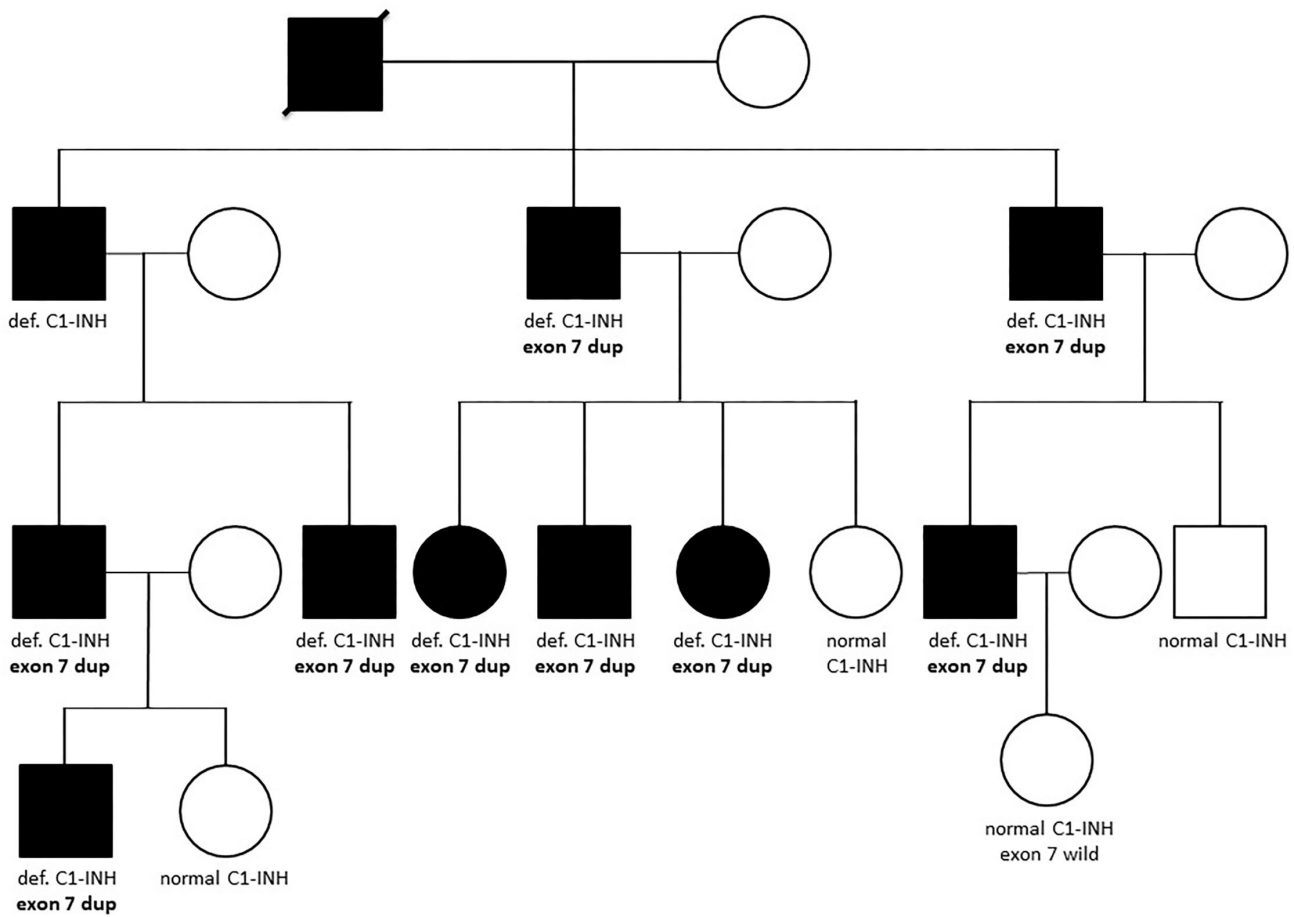
Family tree of a patient with the novel *SERPING1* exon 7 duplication. Affected members of the family are depicted with dark symbols, while all available results of complement and genetic measurements are denoted below the symbols. Def. or normal C1-INH means deficient or normal activity and level of C1-inhibitor, respectively. “Exon 7 dup” refers to the heterozygous carrier state of the duplication of *SERPING1* exon 7, while individuals denoted as “exon 7 wild” does not carry this variation.

In those patients in whom no sequence alteration was found with Sanger sequencing or the pathogenicity of the identified variation was not undoubtedly supported, copy number of *SERPING1* exons was studied by MLPA (Figure 1). A part of our patient group was analyzed previously with Southern blot technique combined with relative quantification of *SERPING1* exons using real-time PCR and SybrGreen detection (24), and the MLPA provided concordant result in each case. Five different deletions involving one or more *SERPING1* exons were identified all of which were reported previously in C1-INH-HAE patients (Table 5). MLPA results showed three copies of exon 7 in case of each available affected members of a large family (Figure 2) and as this mutation was not reported previously, patients from this family were further investigated in order to verify the duplication of exon 7 with an independent method. Since MLPA indicated an extra copy of the site recognized by the exon 7 probe, forward and reverse PCR primers (listed in Table 1) were designed for this region to amplify the sequence between the duplicated exon 7 probe binding sites. Applying this PCR, an app. 4 kb product was generated in samples of each affected individual of the corresponding family but not from healthy controls (data not shown). Complement measurements showed deficient C1-INH activity and level in the available members with edematous symptoms, while normal complement results were obtained from the analyzed healthy relatives (Figure 2).

**Figure 3:**
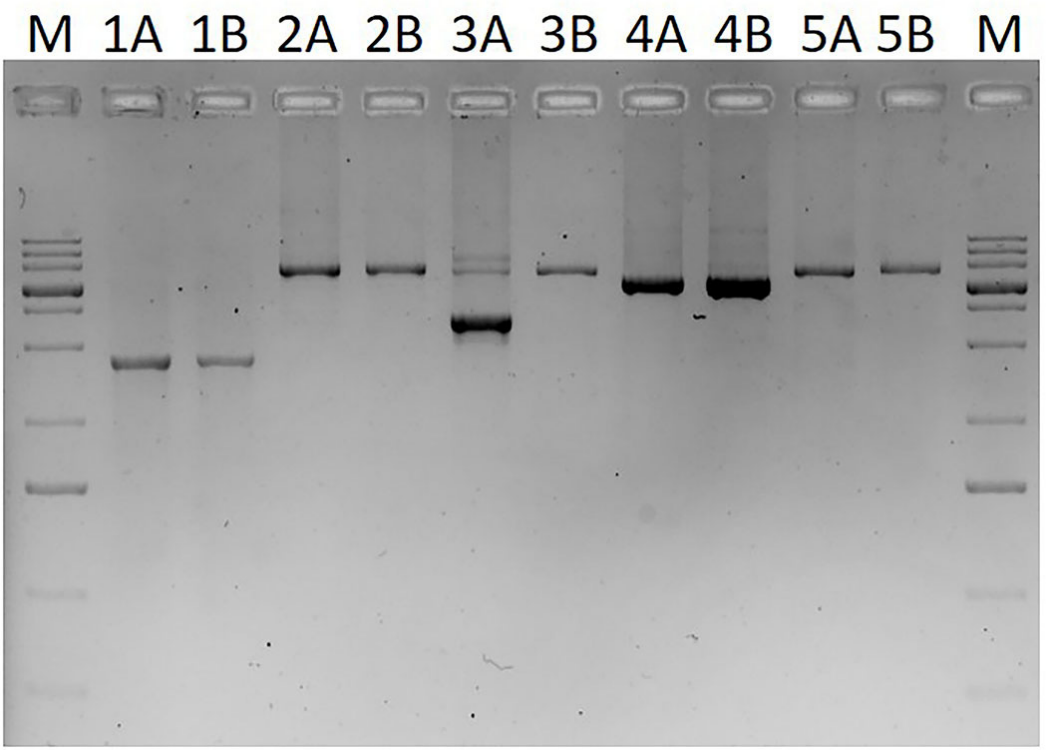
Image of agarose gel (1%) electrophoresis of long-range PCR products covering the whole *SERPING1* gene. M: GeneRuler 1 kb plus DNA ladder (Thermo Scientific, Waltham, MA, USA). PCR products in lines involve the following exons: lines 1: exons 1–3 (from the binding site of the MLPA probe specific for exon 1), lines 2: exons 3–5, lines 3: exons 5–7, lines 4: exons 7–8, lines 5: exon 8 (plus an extra 1375 bp downstream from the 3'-end of exon 8). Samples marked with A (1A−5A) are derived from a C1-INH-HAE patient, samples with B (1B−5B) are from a healthy control.

In the case of two families, MLPA showed two copies for each studied exon and a normal sequence was retrieved from Sanger sequencing of exons and exon–intron boundaries in the patients. However, these methods have limitations and may overlook certain mutations, such as deletions that does not involve the recognition site of any of the MLPA probes or mutations that affect one or both of the PCR primer binding sites. To overcome this problem, a series of long-range PCRs was applied that cover the whole *SERPING1* gene with 2–7 kb long products. As Figure 3 shows an extra, smaller band was observed in case of the long PCR amplifying the region of exon 5–7 and sequencing of this product revealed that the patient carries a 2606 bp long deletion including the last 91 bp of exon 6 and 2515 bp of intron 6 (c.939_1029+2515del). This particular long-range PCR was applied to analyze the available family members of the patient showing that his affected father also carried this deletion but PCR performed from the samples of his symptom-free mother and aunt (the father's sister) revealed only wild-type products. In agreement with this, deficient C1-inhibitor activity and level were detected only in the index patient and his father.

In the remaining one family, a deep intronic *SERPING1* variation (c.1029+384A>G) was detected by a targeted next-generation sequencing (NGS) panel as reported previously (34). In order to verify this variant with Sanger sequencing and to screen for its carrier state in new patients, a new reverse primer was designed for the previously used forward one and since then applied in the diagnostic work-up (Table 1).

Besides the mutation identified as pathogenic, two rare variations were detected in two families, a sense variant (p.Leu251=) in the two affected members of a family with exon 4 deletion and a rare missense polymorphism (p.Arg366His) in a patient without available family members, who also carried the p.Glu85^*^ nonsense mutation (3).

As a probable causative variation was detected in each of the studied Hungarian families, the diagnosis based on complement measurements was verified by genetic testing in each of the patients. Knowledge of the *SERPING1* mutation allowed the verification of C1-inhibitor deficiency in 24, at the time of the analysis yet symptom-free individuals. These subjects included 11 children under the age of 1 year, when complement levels (especially C4) may be lower than the normal adult levels in healthy children as well (36). Among them, 5 were newborns, in whom the analyzed DNA was isolated from the umbilical cord. Moreover, exclusion of C1-INH-HAE was achieved from the umbilical cord DNA of 4 offspring including two cases with ambiguous complement results (below normal C4 and C1-INH level with normal C1-INH function).

## Discussion

Based on the current guidelines, molecular genetic testing is not obligatory as a first-line diagnostic approach in case of patients with angioedema, as most of the cases can be diagnosed based on the clinical picture, complement laboratory findings (functional and antigenic levels of C1-inhibitor, concentrations of C4, C1q, and autoantibodies against C1-inhibitor) as well as family history (if available) (9). However, it is still of high importance to report the identified variations that (1) have a clear damaging effect on protein function/level, or that (2) are segregating with the disease or that (3) are confirmed to be pathogenic by functional studies, as their publication or inclusion in disease-specific databases may help further investigators to decide about their pathogenicity.

Here, we report the mutational spectrum of a large cohort of Hungarian C1-INH-HAE patients diagnosed in the Hungarian Angioedema Center of Reference and Excellence, the national center caring all Hungarian C1-INH-HAE patients. This is an update of the whole Hungarian cohort diagnosed in the past almost 50 years including families with previously published genetic background (3, 24, 32–34, 37, 38), but extended with further family members and also newly diagnosed patients with the identified *SERPING1* mutations. By applying conventional molecular genetic methods such as Sanger sequencing and MLPA, a pathogenic *SERPING1* variation could be explored in almost all families: C1-INH deficiency was caused by a missense or nonsense mutation in 47.1% (32/68), by a small deletion, insertion, or delins in 26.5% (18/68), by a large deletion or duplication in 13.2% (9/68) or by an intronic variation in 10.3% (7/68) of the analyzed pedigrees.

Among missense variations one novel was identified, an adenine to cytosine substitution in exon 4, causing a tyrosine to serine change at codon 199 of the C1-inhibitor protein. This novel substitution was not found in international databases studying large populations with different ethnicity (gnomAD: https://gnomad.broadinstitute.org/; 1,000 Genomes project: https://www.internationalgenome.org/; NHLBI GO Exome Sequencing Project: https://evs.gs.washington.edu/EVS/). Applying five prediction tools to assess its pathogenicity, ambiguous results were retrieved, however, with a dominance of damaging predictions (4 out of 5). The pathogenic role of this p.Tyr199Ser variation is supported by several factors, one of which is that the two carrier children in the family have recurrent angioedema attacks and their complement parameters are typical of C1-INH-HAE. Coinheritance of deficient C1-inhibitor level and activity with the mutation was also observed as the mother with insufficient level and activity of C1-inhibitor is a mutation carrier as well. However, segregation of the mutation with symptoms was not clearly confirmed as she only once had an attack of suspicious abdominal pain, but never experienced subcutaneous edematous episodes. The pathogenicity of the identified p.Tyr199Ser variation located in the helix-rich region of the serpin domain is also underpinned by a previous study that described a pathogenic tyrosine–asparagine change at this position (p.Tyr199Asn), indicating the importance of the tyrosine at codon 199 (7).

Another novel single nucleotide variation, the insertion of an adenine (c.812dup) was detected in a patient without available family members who had decreased level of C1-INH and C4 as well as deficient functional activity of C1-INH. The pathogenicity of this mutation is clear, as by the duplication of one base it shifts the reading frame and results in the generation of a premature termination codon in the formed mRNA.

In our C1-INH-HAE cohort, five gene rearrangements were identified by MLPA: four previously published large deletions involving 1 to 8 exons and a duplication of exon 7 which was not reported previously. The presence of this novel copy number variation was verified by long-range PCR in each affected individual of the corresponding family; moreover, this large pedigree allowed the detection of clear cosegregation of the mutation with C1-INH-HAE symptoms and deficient complement levels. Besides, amplification of an app. 4 kb product by applying forward and reverse primers specific for the MLPA exon 7 probe hybridization site supported the close (within gene) location of the extra copy of this site.

After performing the analysis of *SERPING1* gene with Sanger sequencing and MLPA, two families (2.9% of the pedigrees analyzed by our group) still remained in which no mutation could be identified. This is in agreement with previous studies (3, 27) and a meta-analysis of Ponard et al. who described that no *SERPING1* gene alterations could be identified using Sanger and MLPA methods in 24 families fulfilling the clinical and laboratory criteria of C1-INH-HAE (24/379; 6.3%) (2). Patients in these two families were further analyzed by a series of long PCRs that was developed in order to detect medium-sized alterations that involve only a small part of an exon and not affect hybridization sites of the MLPA probes. These kinds of mutations are not detected by MLPA and if the binding site of the forward or reverse PCR primer is also deleted, it won't be detected by Sanger sequencing either as only the wild-type allele will be amplified from the other chromosome. Long-range PCR is a widely accepted method to investigate large gene rearrangements, and was applied by several groups in the genetic diagnosis of HAE as well (3, 27, 39). These PCRs were usually designed to amplify extra-large products (sometimes more than 10–15 kb) in order to capture large deletions, or even deletion of the whole gene; however, capacity of these methods to detect deletions affecting only a few hundred or thousand nucleotides is limited. By applying the MLPA technique, we were able to detect large rearrangements; therefore, in this study, we rather focused on smaller deletions and applied PCR primers to amplify 2–7 kb products. With this method, a partial deletion was detected involving the last 91 bp of exon 6 and 2,515 bp of intron 6 in the two symptomatic cases but not in two healthy members of the corresponding family. Functional relevance of this mutation is obvious either by causing the deletion of 91 bp from the coding sequence resulting in reading frameshift or by abolishing the exon–intron boundary impairing correct splicing. Moreover, the clear cosegregation of this mutation with deficient C1-INH function and level as well as edematous symptoms also confirms its pathogenicity.

In one family, the standard approaches failed to uncover the disease-causing *SERPING1* alteration; therefore, a NGS platform targeting the entire *SERPING1* gene was performed that resulted in the identification of a deep intronic variation (c.1029+384A>G) as published in details previously (34). Intronic regions were initially considered to be mostly nonfunctional and variations found deep within the introns (i.e., in a distance of more than 100 base pairs away from the exon–intron boundaries) were disregarded as pathogenic alterations having any functional consequences. However, with the advent of whole-genome sequencing a few novel deep intronic variants were identified in *SERPING1* that showed a clear association with the symptoms of C1-INH-HAE based on recent studies, supporting the functional importance of the intronic sequences (6, 40). As Hujová's study nicely presented the *SERPING1* c.1029+384A>G mutation results in *de novo* donor splice site creation and subsequent pseudoexon inclusion (6).

In each affected family of our C1-INH-HAE cohort of almost 200 subjects, we were able to detect a variant that was classified as “pathogenic” or “potentially pathogenic” based on the criteria of ACMG (16). Identifying the disease-causing *SERPING1* variation could be highly useful in those cases where the clinical diagnosis of C1-INH-HAE is highly suspected but the complement test results are inconclusive. This occurs frequently before or upon the first presentation of HAE-like symptoms (such as unexplained gastrointestinal symptoms) in children with a positive family history of C1-INH-HAE, where the functional and antigenic C1-INH levels are not reliable parameters of C1-INH-HAE before the first year of age, as their reference ranges are significantly lower compared to the adult reference ranges (8, 9, 41). Knowledge of the causative mutation allowed us to perform genetic analysis immediately after birth from umbilical cord samples of newborns with a parent suffering from C1-INH-HAE. In five cases, sequencing or copy number analysis of the corresponding exon(s) indicated inheritance of the *SERPING1* mutation, while it excluded C1-INH-HAE in four newborns (including two cases with uncertain complement results). Besides, determination of the mutation carrier state verified the diagnosis in further 6 children under the first year of age when complement measurements may be inconclusive.

Considering the routinely used molecular genetic approaches such as bidirectional sequencing of *SERPING1* exons and the exon/intron boundaries along with the copy number determination of *SERPING1* exons, these are able to explore most of the disease-causing variations in C1-INH-HAE patients. However, in those families where complement laboratory studies suggest the diagnosis of C1-INH-HAE but no *SERPING1* mutation can be identified by the conventional methods used in a diagnostic laboratory, or in case the detected missense *SERPING1* variation is supposed to be benign according to previous publications, databases or *in silico* prediction tools, further unique techniques should be applied in order to explore the pathogenic alteration in the background of the disease. Discovering *SERPING1* variations in C1-INH-HAE patients and reporting data that contribute to the correct interpretation of the pathogenicity of these variations are of utmost importance since knowledge of the genetic background may support the establishment of the correct and early diagnosis of C1-INH-HAE promoting the patient's proper treatment, prognosis, and quality of life.

## Data Availability Statement

The original contributions presented in the study are included in the article/supplementary material, further inquiries can be directed to the corresponding author.

## Ethics Statement

The studies involving human participants were reviewed and approved by Medical Research Council of the Ministry of Human Capacities in Hungary (approval number: 55381-1/ 2015/EKU) and the institutional review board of the Semmelweis University, Budapest. Written informed consent to patients, or participate in this study was provided by the participants' legal guardian/next of kin.

## Author Contributions

ES, DC, and ÁS: performed genetic analyses, participated in writing, and technical editing of the manuscript. NA: collected patient's data. LV: managed complement measurements. HF: provided and cared for study patients. The final version of the article was read and approved by all the contributors. All authors contributed to the article and approved the submitted version.

## Funding

The study was supported by NKFI 124557.

## Conflict of Interest

The authors declare that the research was conducted in the absence of any commercial or financial relationships that could be construed as a potential conflict of interest.

## Publisher's Note

All claims expressed in this article are solely those of the authors and do not necessarily represent those of their affiliated organizations, or those of the publisher, the editors and the reviewers. Any product that may be evaluated in this article, or claim that may be made by its manufacturer, is not guaranteed or endorsed by the publisher.

## Abbreviations

ACMG: American College of Medical Genetics and Genomics
CADD: Combined Annotation Dependent Depletion
C1-INH: C1-inhibitor
C1-INH-HAE: Hereditary angioedema due to C1-inhibitor deficiency
HAE: Hereditary angioedema
MLPA: Multiplex ligation-dependent probe amplification
PCR: polymerase chain reaction
SIFT: Sorting Intolerant From Tolerant.
